# Medical News

**Published:** 1851-06

**Authors:** 


### MEDICAL NEWS.

DR. ELISHA BARTLETT has received and accepted the appointment of Professor of Materia Medica and Medical Jurisprudence in the College of Physicians and Surgeons, of New York, vacated by the death of Dr. John B. Beck.

DR. SAMUEL D. GROSS has resigned the Professorship of Surgery in the Medical Department of the New York University.

Dr. ALFRED C. POST has been appointed Professor of Surgery, and Dr. MEREDITH CLYMER, Professor of the Principles and Practice of Medicine in the Medical Department of the New York University.

A RICH SURGEON.—Ralph Fletcher, Esq., a surgeon of some note in Gloucester, England, died lately, leaving a fortune, acquired in the practice of his profession, of *eighty thousand pounds* (about four hundred thousand dollars.)

